# Supplementary figures and images for: Birth cultures: A qualitative approach to home birthing in Chile
Source: PLoS One. 2021 Apr 22;16(4):e0249224. doi: 10.1371/journal.pone.0249224 (PMC8062023; doi:10.1371/journal.pone.0249224)

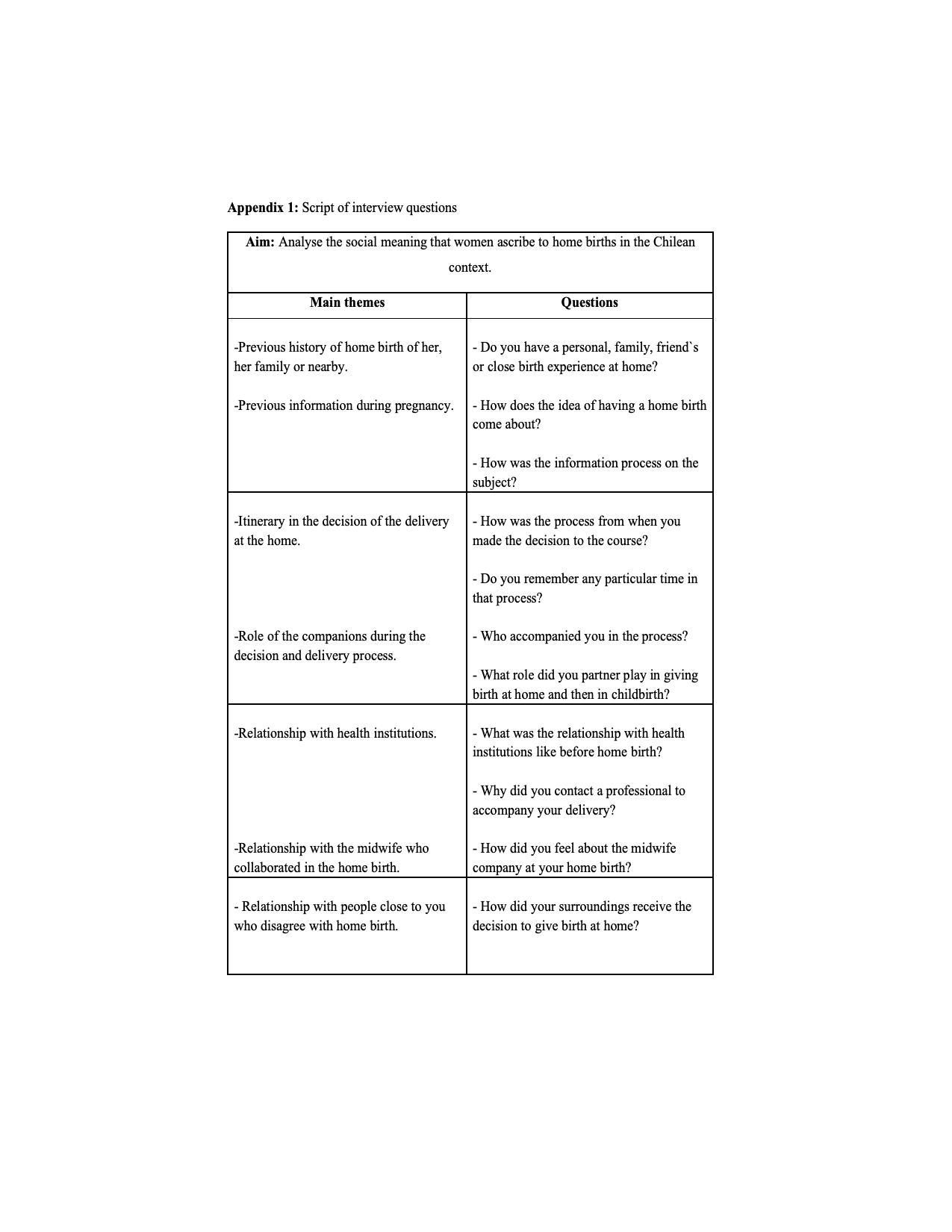

Supplement: S1 Appendix — (TIFF) [file pone.0249224.s001.tiff]
