# Supplementary material for: Birth cultures: A qualitative approach to home birthing in Chile
Source: PLoS One. 2021 Apr 22;16(4):e0249224. doi: 10.1371/journal.pone.0249224 (PMC8062023; doi:10.1371/journal.pone.0249224)
Supplement: Attachment — Submitted filename: Response to Reviewers.docx [file pone.0249224.s002.docx]

Barcelona, January 25^th^, 2021

Dear Academic Editor,

We send you esteemed regards and respectfully submit our rebuttal letter, which includes the following: The Academic Editor’s suggestions, the journal requirements, the reviewers’ suggestions, and the modifications implemented by the article’s authors.

| **Academic Editor** | **Authors** |
| --- | --- |
| A rebuttal letter that responds to each point raised by the academic editor and reviewer(s). You should upload this letter as a separate file labeled 'Response to Reviewers'. | The rebuttal letter has been completed and the modifications suggested by the Academic Editor and the reviewers have been implemented. It is attached as “Response to Reviewers”. |
| A marked-up copy of your manuscript that highlights changes made to the original version. You should upload this as a separate file labeled 'Revised Manuscript with Track Changes'. | The final version is attached, entitled “Revised Manuscript with Track Changes”. |
| An unmarked version of your revised paper without tracked changes. You should upload this as a separate file labeled 'Manuscript'. | The final version of the file is attached, entitled “Manuscript”. |

| **Journal Requirements** | **Authors** |
| --- | --- |
| 1) Please ensure that your manuscript meets PLOS ONE's style requirements, including those for file naming. | Completed |
| 2) Please include your tables as part of your main manuscript and remove the individual files. Please note that supplementary tables (should remain/ be uploaded) as separate "supporting information" files. | Completed |
| 3) Please include additional information regarding the survey or questionnaire used in the study and ensure that you have provided sufficient details that others could replicate the analyses. For instance, if you developed a questionnaire as part of this study and it is not under a copyright more restrictive than CC-BY, please include a copy, in the original language as well as the English version already provided, as Supporting Information. | No questionnaire was used during the study. However, the interview script is attached as Supporting Information. |
| 4) During our internal checks, the in-house editorial staff noted that you conducted research in another country. Please ensure that you have suitably acknowledged the contributions of any local collaborators involved in this work in your authorship list and/or Acknowledgements. | Completed |
| 5) In your Methods section, please provide additional information about the participant recruitment method and the demographic details of your participants. Please ensure you have provided sufficient details to replicate the analyses such as: a) the recruitment date range (month and year), b) a statement as to whether your sample can be considered representative of a larger population, and c) descriptions of where the research took place. | Completed |
| 6) We note that Figure 1 in your submission contains map images which may be copyrighted. All PLOS content is published under the Creative Commons Attribution License (CC BY 4.0), which means that the manuscript, images, and Supporting Information files will be freely available online, and any third party is permitted to access, download, copy, distribute, and use these materials in any way, even commercially, with proper attribution. For these reasons, we cannot publish previously copyrighted maps or satellite images created using proprietary data, such as Google software (Google Maps, Street View, and Earth). | The author’s signed permission to use her figure is attached.  A legend has been included in the figure 1 that mentions said permission and the names the figure’s author. |
| 7) Please amend either the title on the online submission form (via Edit Submission) or the title in the manuscript so that they are identical | Completed |
| 8) Please include captions for your Supporting Information files at the end of your manuscript, and update any in-text citations to match accordingly. | Completed |

| **Reviewer 1** | **Authors** |
| --- | --- |
| One concern I have is that you seem to accept the claim that medicine has made that hospitalization is responsible for the lowered maternal and infant morbidity and mortality rates historically, and the data does not support that. Over time, parity decreased, age at first births increased, some general public health measures occurred -- all of which improved infant and maternal health. Where we actually have the data, we have been able to see that hospitalization and the destruction of midwifery care harmed not improved outcomes. That was my main concern. | The wording of the Results section has been modified (p. 20) and a paragraph has been added to the Discussion section (p. 33) clarifying that lowered maternal and infant mortality rates are not due exclusively to the hospitalization of childbirth. |

| **Reviewer 2** | **Authors** |
| --- | --- |
| Title: As SRQR guidelines, it would be useful to include the qualitative approach in the title. | The qualitative approach has been added to the title. |
| Abstract: An abstract is included with clear aim, methods. Enumeration of categories/subcategories in “Findings” seems a bit confusing. Was there one major theme “home birth journey” with two subthemes: “making the decision to give birth at home” (Why do I need to give birth at home? the people around me don’t support me), and “giving birth” (Shifting emotions during home birth; I (don’t) want to be alone). | The explanation of the categories in the Results section has been clarified. |
| Introduction The introduction provides a good overview of birth cultures, feminists approach to motherhood and rational for research. Some citations need to be amended to Vancouver style. | The entire text has been reviewed to ensure compliance with Vancouver style. |
| Methods The methods section describes a qualitative approach (using a phenomenological paradigm and feminist perspective) and rational for this. Context, setting, participant selection is generally well described. Perhaps more contextual factors could be described (e.g., maternity health system, general population health, needs). Recruitment process is not very detailed (e.g., written info provided? who gained consent, timeline, how many weeks after birth were women interviewed? how participants were selected among the 100 interested?). Why were those baseline characteristics selected? | A new subsection, “Healthcare and Birthing in Chile”, has been created. In this section, the social facets of healthcare associated with home births in Chile have been explored.  Participant recruitment and selection criteria have been addressed in greater detail and a table has been added to help explain the interview timeline.  In the Ethical Considerations section, the way in which the first author of the manuscript performed informed consent and in-person interviews has been described. |
| The approval ethical statement needs to be updated in the methods section (it is XXX at the moment). In the submission form authors mention ethical approval from the University of Barcelona, Spain. Since this study was conducted in Chile, it is surprising not to have any Chilean regulatory ethical body review or approval. No author has any Chilean affiliation and no Chilean organization is acknowledged. Could this have any implications for research and contextualization of findings? It would be useful for authors to clarify this. | In the Ethical Considerations section, the Master’s program in gender studies and psychosocial intervention at Universidad Central de Chile has been mentioned as the academic entity that supervised and collaborated on the logistical aspects of empirical work carried out in Chile.  In the Acknowledgements section, said Master’s program has been mentioned again and thanked for their support and collaboration. |
| In terms of data collection: How were interviews conducted (face to face, online?)? Who did the interviews/researcher’s characteristics and reflexivity? Data analysis and different steps of thematic analysis are very well described. It would be useful to know if any specific techniques were used to guarantee rigour/trustworthiness of data. | In the Data Collection section, the way in which the first author of the manuscript carried out the in-person interviews has been explained in greater detail.  In the Methodological Rigor section, reflexivity criteria have been described in detail together with the rest of the criteria that shape the methodological rigor of the research performed. |
| Results/Findings Having here the table with characteristics of interviews of women will be very useful, as well as the timeline for interviews (X days/weeks/months after birth). A summary of 1-2 lines of baseline characteristics will be useful. | Some participant characteristics have been outlined briefly in the Results section. |
| It feels that some statements in these sections are not findings from this study explicitly and should perhaps fit better within the discussion section and referenced (e.g., page 15: The infamy surrounding home births is no coincidence. Let us recall that in olden days, women gave birth inside their homes, attended to by a birth assistant. However, the modernization ushered in by the industrial revolution has resulted in the institutionalisation of birth….; and women have gone from laboring in the privacy of their homes to inside hospitals. This has led to a lower maternal and neonatal mortality rate;…; page 19: “Obstetric violence encompasses a series of practices and attitudes such as physical aggression toward pregnant women, verbal abuse, acts of coercion, infantilisation and the performance of unjustified caesarean sections…” ; page 22: “The instant the baby is pushed out during a vaginal birth is regarded as a culminating moment, the climax of a series of stages that, from a physiological point of view, correspond to neurohormonal and physical events”; page 24: as stated by Le Breton [37], emotions are ‘a real or imaginary, past, present….etc) | The wording and description in the indicated paragraphs have been corrected, and some theoretical reflections that do not correspond to the Results section have been deleted. |
| Discussion is well presented with links to empirical data and good refs (again citations and ref need to be updated to Vancouver style). P28. “It is evident that significant scientific evidence exists supporting home birth. However, healthcare professionals still hesitate to recommend it as a safe option” Does this refer to Chile? It feels this is hugely dependant on context. Are there limitations of the study that need to be considered? (e.g., sample size, lack of data on baseline socio-demographic characteristics) | The citations have been corrected to comply with Vancouver style.  On page 33, an explanation that the vast amount of existing scientific evidence concerning home births exists at the international level has been inserted.  At the end of the Discussion section, the study’s main limitations have been stated in detail. |

| **Reviewer 3** | **Authors** |
| --- | --- |
| Introduction This study has a strong theoretical foundation, but the authors don't make a clear case for the need for the current research. What gap in knowledge is it filling? Minor note: Robbie Davis-Floyd is a woman. The sentence citing reference 15 should read "for her part..." There are also a few sentences in the introduction that are not clear: The dominant structures crossed by economic and political models that guide the behaviors of society, have precarious and violated the collective dynamics surrounding motherhood and parenting (Missing a word?) Situation that worsens in the puerperium with breastfeeding [13] (Incomplete sentence) However, not all advocates are homogenous. (Do you mean not all contexts are homogeneous?) | The justification for addressing this particular problem has been improved, and the contribution that this research makes to the international and national scientific communities has been explained.  The wording of the sentence beginning “For his part…” has been modified.  The wording of the paragraph beginning “The dominant structures…” has been modified.  The sentence beginning “Situation that worsens in…” has been deleted because it did not fit the context.  The explanation of difference among countries as heterogenous contexts has been improved. |
| Methods Can you provide more justification for the geographical diversity of the sample, and how you expected this to affect your results? The availability of health care is very different across the three sample regions. Did you expect the themes to be different? Please provide more detail regarding sampling and inclusion criteria. You state that you had 110 potential participants, but you only interviewed 30. Were any of them excluded for reasons other than the inclusion criteria? Also, why 30 participants? Did you think you would reach saturation with 30? Did you actually reach saturation? Was 30 selected for practical considerations? Please provide more description in the text regarding the two "complementary processes" involved in the interview script development. These processes are not clear. From the figure, it looks like you identified main themes from a literature search-- that's one process. What was the second? | The importance of incorporating women from different regions in Chile into the study has been incorporated.  The feasibility criteria applied during the qualitative research process as related to sampling and inclusion has been described. The consideration of feasibility and practical aspects of carrying out this research project enabled both the researcher and the interviewee to determine their availability for participating in the study. Relevant aspects included geographical distance, mobility and availability of time (most participants had given birth no more than one year previously).  The explanation of the processes by which the interview questions were drafted has been improved. |
| Results There are several paragraphs in the results section that are more appropriate for the discussion. The results section should describe your findings, not describe related literature. | Some paragraphs have been deleted and others have been moved to the Discussion section. |
| Discussion and Conclusions These were strong but could be enhanced by incorporating some of the content that was prematurely included in the results. | Some of the Discussion section paragraphs have been modified and others from the Results section have been added. |
