# Supplementary material for: Birth cultures: A qualitative approach to home birthing in Chile
Source: PLoS One. 2021 Apr 22;16(4):e0249224. doi: 10.1371/journal.pone.0249224 (PMC8062023; doi:10.1371/journal.pone.0249224)
Supplement: Attachment — Submitted filename: Response to Reviewers.docx [file pone.0249224.s003.docx]

Barcelona, March 04^th^, 2021

Dear Academic Editor,

We send you esteemed regards and respectfully submit our second rebuttal letter, which includes the following: The Academic Editor’s suggestions, the reviewers’ suggestions, and the modifications implemented by the article’s authors.

| **Academic Editor** | **Authors** |
| --- | --- |
| A rebuttal letter that responds to each point raised by the academic editor and reviewer(s). You should upload this letter as a separate file labeled 'Response to Reviewers'. | The rebuttal letter has been completed and the modifications suggested by the reviewers have been implemented. It is attached as “Response to Reviewers”. |
| A marked-up copy of your manuscript that highlights changes made to the original version. You should upload this as a separate file labeled 'Revised Manuscript with Track Changes'. | The final version is attached, entitled “Revised Manuscript with Track Changes”. |
| An unmarked version of your revised paper without tracked changes. You should upload this as a separate file labeled 'Manuscript'. | The final version of the file is attached, entitled “Manuscript”. |

| **Reviewer 3** | **Authors** |
| --- | --- |
| The additions regarding the context and sampling decisions greatly strengthen the paper. I have one small note regarding an addition. This sentence is a bit confusing: Of those, 33% entailed caesarean sections performed via the public system and 63% took place within the private system, positioning Chile as a country with one of the world’s highest rates of C-sectioning [31]. I recommend rephrasing for clarity: Within the public system, 33% of births are by cesarean delivery and 63% of those in private system are by cesarean, positioning Chile as a country with one of the world’s highest rates of C-sectioning [31]. | Completed  The sentence has been modified according to the suggestion of the Reviewer #3.  We have added this modification suggested by Reviewer 3 in the manuscript that already contained the previous modifications. (See page n.7). Please, tell us if we should previously accept the modifications of the first revision and add "with Track Changes" only this modification suggested in the current revision. |
